# Features of Obstructive Sleep Apnea in Children with and without Comorbidities

**DOI:** 10.3390/jcm12062418

**Published:** 2023-03-21

**Authors:** Eusebi Chiner, Jose N. Sancho-Chust, Esther Pastor, Violeta Esteban, Ignacio Boira, Carmen Castelló, Carly Celis, Sandra Vañes, Anastasiya Torba

**Affiliations:** Pulmonology Department, Multidisciplinary Sleep Clinic, San Juan de Alicante University Hospital, 03550 Alicante, Spain

**Keywords:** childhood sleep apnea, adenotonsillar hypertrophy, concomitant disease, predisposing factors

## Abstract

Background: To compare the clinical and polysomnographic features of obstructive sleep apnea (OSA) in children with adenotonsillar hypertrophy (Group A) and comorbidities (Group B). Methods: A five-year prospective study using nocturnal polysomnography before and after treatment. Results: We included 168 patients: 121 in Group A and 47 in Group B, with differences in age (6.5 ± 3 vs. 8.6 ± 4 years; *p* < 0.001), body mass index (BMI) (18 ± 4 vs. 20 ± 7 kg/m2; *p* < 0.05), neck circumference (28 ± 4 vs. 30 ± 5 cm; *p* < 0.05), and obesity (17% vs. 30%; *p* < 0.05). Group B patients were more likely to have facial anomalies (*p* < 0.001), macroglossia (*p* < 0.01), dolichocephaly (*p* < 0.01), micrognathia (*p* < 0.001), and prognathism (*p* < 0.05). Adenotonsillectomy was performed in 103 Group A patients (85%) and 28 Group B patients (60%) (*p* < 0.001). In B, 13 children (28%) received treatment with continuous positive airway pressure (CPAP) and 2 (4%) with bilevel positive airway pressure (BiPAP), compared with 7 in Group A (6%) (*p* < 0.001). Maxillofacial surgery was more common in Group B (*p* < 0.01). Clinical and polysomnography parameters improved significantly in both groups after therapeutic intervention, though Group A showed better results. Conclusions: Obesity and facial anomalies are more frequent in childhood OSA patients with comorbidities, who often require combination therapy, such as ventilation and surgery.

## 1. Introduction

Childhood obstructive sleep apnea (OSA) has a worldwide prevalence of 1–5%, peaking in children aged 2 to 8 years and increasing to 7–16% when snoring in children aged 6 months to 13 years is also considered [1,2].

As in adult OSA, the main symptom of childhood OSA is snoring, frequently accompanied by apnea; however, children also show other manifestations, such as mouth breathing, failure to thrive, enuresis, and neuropsychic disorders [3,4]. Based on these varied symptoms, we can distinguish two phenotypes: (A) adenotonsillar hypertrophy and (B) concomitant diseases or comorbidities (with or without adenotonsillar hypertrophy), including facial anomalies, neuromuscular diseases, and complex syndromes (Down’s syndrome, Prader–Willi syndrome, etc.).

Patients in both groups have reduced upper airway caliber and dynamic alterations, and although a clear relationship exists between adenotonsillar hypertrophy size and the apnea–hypopnea index (AHI), no study has revealed intervening factors or shown why adenotonsillectomy has different effects in OSA patients and controls [2]. In addition, obesity is a growing problem in European countries and is a known predisposing factor in adults [4,5,6]. Obesity alone can lead to OSA, and may be associated with adenotonsillar hypertrophy and a higher risk of residual disease after adenotonsillectomy in children. OSA is the sleep disorder most closely associated with cardiovascular consequences, primarily high blood pressure [7].

Management of childhood OSA can be complex, particularly in patients with comorbidities, as they require diagnostic tests at onset and during the course of the condition [6]. The current gold standard for OSA diagnosis is polysomnography [8,9].

First-line treatment consists of adenotonsillectomy, since adenotonsillar hypertrophy is found in most patients [10]. Nonetheless, OSA often persists after surgery and care providers must consider alternative and/or complementary treatments, such as intranasal corticosteroids or montelukast [11,12,13]. Residual OSA may require simple or combined treatment; the latter could include a hypocaloric diet, continuous positive airway pressure (CPAP), myofunctional therapy, or rapid maxillary expansion. Treatment should be tailored to individual patients, as no single strategy suits all [1,13].

Most published studies have analyzed single risk factors [13]; none has compared different predisposing conditions, treatment, and evolution in a pediatric population. We hypothesized that the clinical and polysomnographic features of childhood OSA may differ in children with adenotonsillar hypertrophy versus children with comorbidities, and that a more complex long-term strategy may be required to treat the condition. The aim of our study was to evaluate and compare the features of childhood OSA in the two populations.

## 2. Materials and Methods

### 2.1. Study Design and Population

We performed a five-year prospective cohort study in people aged under 14 years who were seen in the Pulmonology Office of San Juan de Alicante University Hospital. We included individuals with suspected OSA, with or without neuropsychiatric or physical daytime symptoms. We excluded patients who refused diagnostic tests or with polysomnography results deemed technically invalid (recording time < 4 h).

### 2.2. Clinical Protocol

We collected information from parents/guardians regarding snoring, apnea, nocturnal shortness of breath, restless sleep, nocturnal sweating, enuresis, nasal obstruction, rhinorrhea, mouth breathing, hearing problems, recurring infections, wheezing, heartburn, headaches, hyperactivity or attention deficit, apathy, shyness, drowsiness, poor academic performance, failure to thrive and other disorders, including poor appetite and polyphagia. These data were collected through a standardized data collection form.

We examined the oral cavity of patients to determine Mallampati score, degree of adenotonsillar hypertrophy on the Brodsky scale, and palatal dimensions. We also recorded craniofacial morphological characteristics, height, weight, body mass index (BMI), and BMI percentile. Using the growth charts published by the Centers for Disease Control and Prevention and the American Academy of Pediatrics, we calculated BMI using the formula weight in kilograms divided by height in meters squared, and classified the values into age and sex-specific percentiles. Percentiles over 95 were considered to represent obesity [14].

### 2.3. Diagnostic Procedure

OSA diagnoses were established through polysomnography (Sleep Lab, Jaeger–Viasys^®^, Hoechberg, Germany), which monitored sleep variables—through electroencephalography (C3-A2), electrooculography, and chin electromyography—as well as the following respiratory variables: nasal airflow through cannula, respiratory impedance with uncalibrated thoracoabdominal bands, and oxygen saturation. We also monitored snoring, body position, and transcutaneous carbon dioxide, and performed electrocardiography and tibialis anterior electromyography.

Apnea was defined as a reduction of more than 90% in amplitude of the oronasal flow signal, with or without microarousal or desaturation, and hypopnea was defined as between 30% and 90% reduction with microarousal and/or 3% desaturation. Respiratory effort associated with microarousal was defined as a period of limited flow lasting between 10 s and 2 min and ending with microarousal. The total number of events of apnea, hypopnea, and respiratory effort associated with microarousal was divided by the total sleep time to obtain the apnea–hypopnea index (AHI). The sleep stages were classified in accordance with international guidelines [15,16,17].

Patients underwent polysomnography in the sleep unit in an isolated single bed. They were supervised by a nurse and accompanied by a family member. OSA diagnosis required an AHI of at least 3/h 18 and severity was classified as follows: mild 3–5/h; moderate 5–10/h; and severe more than 10/h.

### 2.4. Patient Groups

After assessing the whole sample, we compared the gender, anthropometric measurements, clinical and polysomnographic features, and evolution of the condition in two groups of patients.

Group A included patients in whom the predominant alteration related to OSA was adenotonsillar hypertrophy.

Group B included patients in whom other comorbidities were the main factors associated with OSA. These comorbidities could include a wide spectrum of severe facial anatomical defects or also marked obesity.

### 2.5. Therapeutic Protocol

After diagnosis, some patients were referred to the otorhinolaryngology department for adenotonsillectomy, and others to maxillofacial surgery. Conservative treatment consisted of montelukast (4–5 mg/day for at least 6 months) combined with dietary, behavioral, and postural modifications. If continuous positive airway pressure (CPAP) was considered, an auto CPAP titration study was performed as part of an adherence program [18]. If bilevel positive airway pressure (BiPAP) was considered, a manual titration was performed to set optimal pressures.

Between 6 and 12 months after surgery, patients underwent follow-up polysomnography. When this was not possible, symptoms were assessed through a clinical interview. We defined improvement as more than 50% reduction in baseline AHI, and recovery as reduction to normal levels.

### 2.6. Sample Size Calculation

Assuming a statistical power of 80%, an alpha error of 5%, and a loss to follow-up of 10%, in concordance with previous studies and owing to the distribution of patients (two-thirds in Group A and one-third in Group B), we estimated that at least 101 patients would be required in Group A and 30 in Group B to detect a statistically significant difference in proportions between the two [2,4]. Sample size calculation was adjusted for gender in each group.

### 2.7. Statistical Analysis

An anonymized database was created using a measure of central tendency for the numerical variables, (mean ± standard deviation), and frequency distribution for categorical variables. We performed a descriptive analysis of clinical features, severity, concomitant disorders, obesity, and therapeutic approach. We used the Chi-square or Fisher’s exact test to compare categorical variables. After applying the Kolmogorov–Smirnov test and checking homogeneity of variance, we compared the numerical variables using the Student’s t-test or the Mann–Whitney test. We used the two-sided paired-samples t-test to compare clinical and polysomnography data before and after treatment in the same group, and the unpaired samples t-test to compare the data of Groups A and B. Values of *p* < 0.05 were considered significant. For all calculations, we used the statistical package IBM SPSS (version 15.0; SPSS; Chicago, IL, USA).

### 2.8. Ethical Aspects

The study was conducted in accordance with the Declaration of Helsinki (updated in Edinburgh), the Council of Europe Convention on Human Rights and Biomedicine, the UNESCO Universal Declaration on the Human Genome and Human Rights, and the provisions governing biomedical research and data protection in Spain. The study was approved by the institutional review board of San Juan de Alicante University Hospital (HUSJ-20-003).

## 3. Results

### 3.1. General Results

Over the five-year study period, we included 168 patients (110 boys and 58 girls) with mean age 7 ± 4 years, BMI 18.7 ± 5.1 kg/m2, BMI percentile 67 ± 37, neck circumference 29 ± 4 cm, and weight percentile 72 ± 43. Forty-two children (25%) were considered obese (BMI percentile ≥ 95). Patients were referred from otorhinolaryngology (39%), pediatrics (35%), pulmonology (21%), neurology (2.5%), and maxillofacial surgery (2.5%). There were 121 children in Group A and 47 in Group B, which included 16 cases of asthma/rhinitis/polyposis; 4 cases each of cystic fibrosis and Down’s syndrome; 3 cases each of gastroesophageal reflux disease and Prader–Willi syndrome; 2 cases each of attention deficit hyperactivity disorder, neuromuscular disorders, epilepsy, morbid obesity, and cerebral palsy; and 1 case each of congenital hypothyroidism, ciliary dyskinesia, Pierre Robin syndrome, Rett syndrome, Rubinstein–Taybi syndrome, Apert syndrome, and congenital atrioventricular block with hearing loss.

In Group A, there were 75 male and 46 female patients. In Group B, there were 35 male and 12 female patients. Gender differences between sexes were not statistically significant (*p* = 0.127).

### 3.2. Clinical Features

The most common nocturnal clinical features in the whole series were snoring (96%), apnea (82%), shortness of breath (70%), and restless sleep (67%). The most common daytime clinical features were recurrent upper airway infections (68%), chronic rhinorrhea (63%), nasal obstruction (58%), daytime mouth breathing (57%), recurrent otitis (29%), daytime sleepiness (26%), failure to thrive (23%), headache (14%), and poor appetite (14%). Neuropsychiatric manifestations were attention problems (33%), poor academic performance (21%), and shyness (11%). Other undefined features reported by parents were weakness, irritability, low activity, polyphagia, poor sleep quality, and recurrent pneumonia.

**Figure 1:**
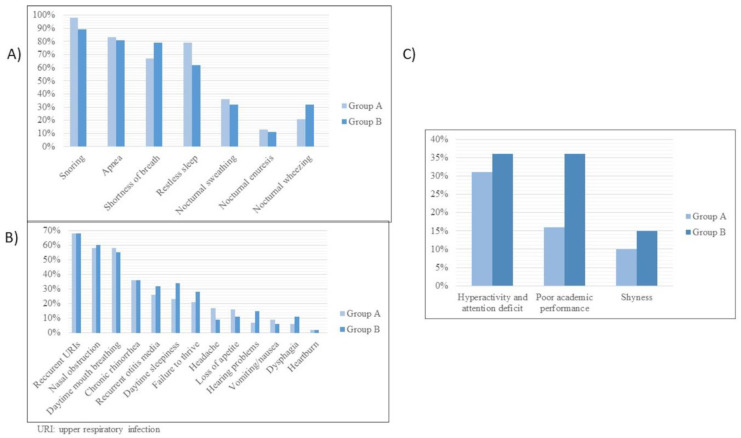
(**A**) Comparison of nocturnal clinical manifestations in Groups A and B. (**B**) Comparison of neuropsychiatric clinical manifestations in Groups A and B. (**C**) Comparison of daytime clinical manifestations in Groups A and B.

Figure 1A–C compares the nocturnal, daytime, and neuropsychiatric features of Groups A and B. The only significant differences were for snoring (98% vs. 89%, *p* < 0.05) and poor academic performance (16% vs. 36%, *p* < 0.01).

Predisposing factors were tonsillar hypertrophy (84%), adenoid hypertrophy (47%), obesity (20%), facial anomalies (17%), high-arched palate (13%), retrognathia (12%), micrognathia (8%), macroglossia (4%), dolichocephaly (4%), and prognathism (1%). Eight patients had prior adenotonsillectomy.

Table 1 compares the anthropometric measurements and predisposing factors of Groups A and B. We found a significantly higher proportion of obesity in Group B (n = 14, 30%) that in Group A (n = 21, 17%) (*p* < 0.05).

### 3.3. Polysomnographic Data

Table 2 presents the polysomnography features of the two groups. Although there were no significant differences in sleep architecture or sleep efficiency, Group B had worse respiratory variables and more microarousals than Group A.

**Figure 2:**
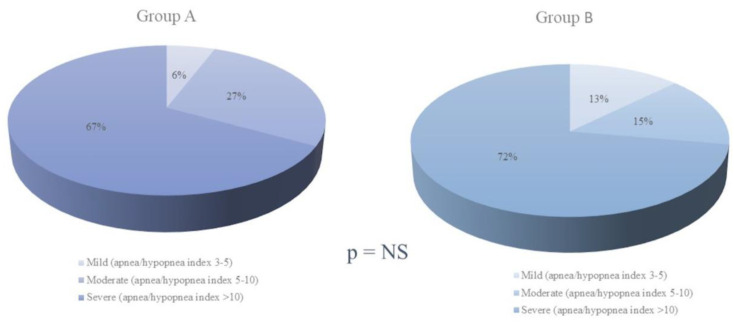
Comparison of obstructive sleep apnea severity measured by the apnea–hypopnea index (AHI) in Groups A and B.

OSA was considered mild in 13 patients (8%), moderate in 40 (24%), and severe in 115 (69%). There were no significant differences in distribution of severity between the groups (Figure 2).

### 3.4. OSA Treatment

Adenotonsillectomy was performed in 28 Group B patients (60%) and 103 Group A patients (85%) (*p* < 0.001). The proportion of children who underwent maxillary surgery or septoplasty was similar in both groups. Other therapeutic measures, such as orthodontic treatment, maxillary distraction, or behavioral therapy, were more common in Group B (n = 5, 11% vs. n = 1, 1%; *p* < 0.01). Thirteen patients in Group B received CPAP (28%), compared with 7 in Group A (6%) (*p* < 0.001). In addition, the two patients treated with BiPAP (4%) were in Group B (*p* < 0.05). Eight Group B patients received conservative treatment (17%), compared with nine from Group A (7.5%). This difference was borderline significant (*p* = 0.07).

**Figure 3:**
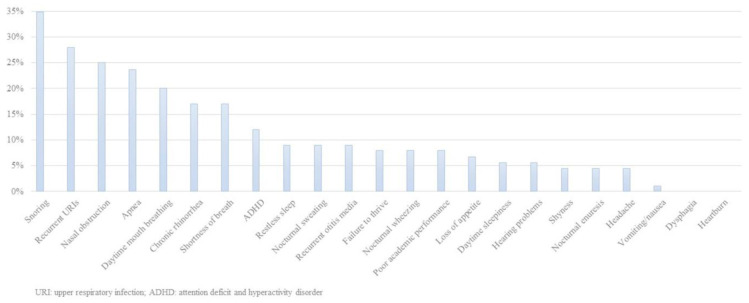
Post-treatment clinical manifestations of apnea–hypopnea syndrome during sleep.

Post-treatment testing was performed in 88 patients (52%)—polysomnography in 47, home respiratory polygraphy in 19, and clinical response follow-up in 22—and we found no differences between the groups in this regard. Both groups had similar nocturnal and daytime symptoms after treatment. All daytime, nocturnal, and neuropsychiatric symptoms had improved significantly in both groups after treatment (*p* < 0.001) (Figure 3).

Table 3 shows the anthropometric and polysomnography values for both groups before and after treatment (polysomnography post-treatment values are for the 47 patients who underwent this procedure).

Of the patients who underwent objective follow-up assessment after treatment, 49 (29%) had a post-treatment AHI of 3 or more: 30 boys and 19 girls; 15 from Group B (32%) and 34 from Group A (28%) (*p* = NS). With an apnea–hypopnea index cutoff point of ≥10, 16 patients (9.5%) were diagnosed with residual OSA: 8 boys and 8 girls; 5 from Group B (11%) and 11 from Group A (9%) (*p* = NS).

## 4. Discussion

Childhood OSA is very common, with an estimated prevalence of 1–5% [1,19,20]. Adenotonsillar hypertrophy is a recognized predisposing factor, but others have been reported, such as craniofacial malformations, neuromuscular disorders, obesity, and Down’s syndrome [20]. Our series contained patients with these conditions.

The predominant features in adults with OSA are obesity, drowsiness, and snoring, while childhood OSA is more frequently associated with failure to thrive, hyperactivity, and other manifestations. Drowsiness was recorded in only 26% of our patients, who were more likely to show failure to thrive, hyperactivity, and attention deficit. OSA has characteristic nocturnal symptoms: 97% of our patients snored, 82% had apnea, and 70% had shortness of breath. The predominant daytime symptoms include recurrent upper airway infection, nasal obstruction, and mouth breathing [4,21], which in our study affected 68%, 58%, and 57% of patients, respectively. Compared with Group B, Group A patients were significantly more likely to snore and have poor academic performance, but no other differences were found regarding night-time or daytime symptoms.

Some of the concomitant diseases in Group B patients may predispose them to OSA. These include asthma, cystic fibrosis, Down’s syndrome, and obesity. Several studies have shown that apnea and snoring are more frequent in people with asthma, and that these symptoms could lead to or aggravate OSA [22]. Other authors have noted that obesity is closely related to wheezing and asthma [23]. One-quarter of all our patients were obese, and this proportion was higher in those with concomitant disease. However, it is worth noting that several patients from both groups had adenotonsillar hypertrophy as well as obesity. Unlike in adult OSA, no clear correlation has been found between AHI and BMI in children under 12 years old [24,25], which leads us to believe that obesity was probably not the most determining factor of OSA in our study.

Children diagnosed with Down’s syndrome are more likely to have OSA owing to a combination of anatomical factors such as macroglossia, midface hypoplasia, and micrognathia, together with obesity and adenotonsillar hypertrophy [26]. Children with Prader–Willi syndrome are also at greater risk of having OSA, associated with obesity, inactivity, drowsiness, and other behavioral disorders [27]. Our study confirms the differences in predisposing factors, as Group B had a significantly higher proportion of macroglossia, facial anomalies, micrognathism, retrognathism, prognathism, and dolichocephaly. In addition, our study demonstrated significantly greater severity in all respiratory variables and significantly more microarousals in children with concomitant disease. We found no differences in other neurological variables, meaning neurological monitoring contributed little to the diagnosis. In contrast, previous findings have highlighted the importance of identifying arousals, as many OSA symptoms may be secondary to sleep disruption, with frequent alterations in sleep architecture increasing the number of arousals and inhibiting deep sleep [23].

While international guidelines recommend polysomnography for the diagnosis of childhood OSA [8], home respiratory polygraphy is emerging as an alternative diagnostic technique in this population [24,28,29,30], although it can only be applied in the hospital setting under current Spanish legislation [1].

Adenotonsillectomy is the treatment of choice for OSA, as it resolves daytime and nocturnal symptoms with an efficacy of 78% [31]. This technique was performed in 60% of Group B compared with 85% of Group A patients, which confirms the increased complexity of treating patients with concomitant disease. This group may require other treatments such as septoplasty, maxillomandibular surgery, maxillary distraction, behavioral therapy, and orthodontic treatment [1]. Indeed, our Group B patients were more likely to receive alternative treatments.

The second most effective therapeutic option for OSA is CPAP, which is more commonly used in children with obesity, neuromuscular disorders, or craniofacial malformations, who may also have adenotonsillar hypertrophy [32]. In our study, there were marked differences between the groups, with a higher proportion of CPAP/BIPAP in Group B.

When treatment is personalized in both groups of patients, it is expected that they will improve equally, although the group with concomitant disease required more frequently CPAP or BiPAP. This would require close monitoring during the growth of these patients, since the underlying disease is persistent and some patients may have to carry these treatments for life.

Several studies have shown that symptoms and cognitive disorders clearly improve in OSA patients after treatment, particularly after adenotonsillectomy [33]. Similarly, in our study, daytime, nocturnal, and neuropsychiatric symptoms improved significantly with treatment. It is also worth noting the significant improvements in anthropometric characteristics, which demonstrates the negative and potentially reversible impact of OSA on child development. Our study highlights the need to distinguish between different phenotypes of childhood OSA and the importance of a multidisciplinary approach, as care providers may need to consider different treatment approaches in the initial clinical assessment [34].

In conclusion, our study showed that childhood OSA is frequently associated with snoring, apnea, shortness of breath, and recurrent upper airway infections. Drowsiness and obesity tend to be associated with concomitant disease. Most cases require adenotonsillectomy, but a third of patients have residual disease and thus need combined treatments, such as CPAP/BiPAP and surgery. This is true particularly in OSA patients with concomitant diseases, who are more likely to have obesity, facial anomalies, and nocturnal hypoventilation. Finally, in our series, the main polysomnography features in childhood OSA were respiratory variables, while neurological factors were largely irrelevant.

## Figures and Tables

**Table 1:** Comparison of age, anthropometric measurements and predisposing factors for sleep apnea–hypopnea syndrome in Groups A and B.

	Group A	Group B	*p* Value
Age (years, mean ± SD)	6 ± 3	9 ± 4	<0.001
Anthropometric measurements (mean ± SD)			
Weight (kg)	28 ± 16	37 ± 21	<0.01
Height (cm)	120 ± 20	130 ± 20	<0.01
BMI (kg/m^2^)	18 ± 4	20 ± 7	<0.05
BMI percentile	66 ± 35	68 ± 42	NS
Neck circumference (cm)	28 ± 4	30 ± 5	<0.05
Predisposing factors (%)			
Tonsillar hypertrophy	93	62	<0.001
Adenoid hypertrophy	49	43	NS
High-arched palate	12	17	NS
Macroglossia	1	11	<0.01
Facial abnormalities	10	34	<0.001
Micrognathism	3	21	<0.001
Prognathism	0	4	<0.05
Retrognathia	8	21	<0.05
Dental malocclusion	3	11	NS
Dolichocephaly	2	11	<0.01
Obesity	17	30	<0.05
Previous tonsillectomy	3	11	<0.05

SD: standard deviation; BMI: body mass index.

**Table 2:** Comparison of polysomnography variables in Groups A and B.

	Group A (Mean ± SD)	Group B (Mean ± SD)	*p* Value
Duration of recording (minutes)	490 ± 64	496 ± 62	NS
Sleep efficiency (%)	87 ± 8	85 ± 9	NS
Stage 1 (%)	13 ± 9	14 ± 10	NS
Stage 2 (%)	30 ± 11	30 ± 13	NS
Stages 3 and 4 (%)	31 ± 14	31 ± 19	NS
REM stage (%)	26 ± 12	23 ± 15	NS
Stage 1 latency	10 ± 23	11 ± 35	NS
Stage 2 latency	12 ± 12	8 ± 7	NS
Stage 3 and 4 latency	138 ± 181	200 ± 196	NS
REM stage latency	70 ± 57	56 ± 50	NS
Microarousal index	14 ± 8	21 ± 18	<0.05
Apnea/hypopnea index	15 ± 9	22 ± 21	<0.05
Oxygen desaturation index	7 ± 8	16 ± 23	<0.05
Baseline oxygen saturation (%)	97 ± 1	95 ± 4	<0.01
Minimum oxygen saturation (%)	79 ± 13	74 ± 17	<0.05
CT90 (%)	4 ± 10	12 ± 20	<0.05

SD: standard deviation; REM: rapid eye movement; CT90: cumulative percentages of time spent at oxygen saturations below 90%.

**Table 3:** Comparison of anthropometric measurements and nocturnal PSG values in Groups A and B before and after treatment.

	Before Treatment (Mean ± SD)	After Treatment (Mean ± SD)	*p* Value
Group A			
Weight (kg)	27 ± 13	31 ± 14	<0.001
Height (cm)	1.0 ± 0.2	1.2 ± 0.2	<0.001
BMI (kg/m^2)^	18 ± 4	19 ± 4	<0.01
Neck circumference (cm)	28 ± 3	29 ± 3	<0.001
Apnea/hypopnea index	17 ± 9	8 ± 7	<0.001
Oxygen desaturation index	7 ± 9	3.7 ± 4	<0.01
Baseline oxygen saturation	97 ± 2	97 ± 1	<0.05
Minimum oxygen saturation	79 ± 15	83 ± 14	NS
CT90 (%)	4 ± 6	7 ± 19	NS
Group B			
Weight (kg)	34 ± 16	36 ± 16	<0.01
Height (cm)	1.2 ± 0.2	1.3 ± 0.2	<0.01
BMI (kg/m^2)^	20 ± 5	20 ± 5	NS
Neck circumference (cm)	29 ± 5	30 ± 5	<0.05
Apnea/hypopnea index	23 ± 19	12 ± 15	<0.05
Oxygen desaturation index	21 ± 24	7 ± 10	<0.05
Baseline oxygen saturation	94 ± 4	96 ± 2	<0.05
Minimum oxygen saturation	71 ± 17	76 ± 14	NS
CT90 (%)	16 ± 21	7 ± 10	<0.05

SD: standard deviation; BMI: body mass index; CT90: cumulative percentages of time spent at oxygen saturations below 90%.

## Data Availability

All data supporting reported results can be found at Pneumology Service, Hospital Universitario San Juan de Alicante (Spain).
